# D-dimer levels on admission and all-cause mortality risk in COVID-19 patients: a meta-analysis

**DOI:** 10.1017/S0950268820002022

**Published:** 2020-09-07

**Authors:** Daniel Martin Simadibrata, Anna Mira Lubis

**Affiliations:** 1 Faculty of Medicine Universitas Indonesia, Jakarta, Indonesia; 2 Division of Hematology and Oncology, Department of Internal Medicine, Dr. Cipto Mangunkusumo National General Hospital, Faculty of Medicine, Universitas Indonesia, Jakarta, Indonesia

**Keywords:** Coronavirus disease, COVID-19, D-dimer, mortality, prognosis

## Abstract

D-dimer level on admission is a promising biomarker to predict mortality in patients with COVID-19. In this study, we reviewed the association between on-admission D-dimer levels and all-cause mortality risk in COVID-19 patients. Peer-reviewed studies and preprints reporting categorised D-dimer levels on admission and all-cause mortality until 24 May 2020 were searched for using the following keywords: ‘COVID-19’, ‘D-dimer’ and ‘Mortality’. A meta-analysis was performed to determine the pooled risk ratio (RR) for all-cause mortality. In total, 2911 COVID-19 patients from nine studies were included in this meta-analysis. Regardless of the different D-dimer cut-off values used, the pooled RR for all-cause mortality in patients with elevated *vs*. normal on-admission D-dimer level was 4.77 (95% confidence interval (CI) 3.02–7.54). Sensitivity analysis did not significantly affect the overall mortality risk. Analysis restricted to studies with 0.5 μg/ml as the cut-off value resulted in a pooled RR for mortality of 4.60 (95% CI 2.72–7.79). Subgroup analysis showed that the pooled all-cause mortality risk was higher in Chinese *vs*. non-Chinese studies (RR 5.87; 95% CI 2.67–12.89 and RR 3.35; 95% CI 1.66–6.73; *P* = 0.29). On-admission D-dimer levels showed a promising prognostic role in predicting all-cause mortality in COVID-19 patients, elevated D-dimer levels were associated with increased risk of mortality.

## Introduction

The World Health Organisation has declared COVID-19, an emerging infectious disease caused by SARS-CoV-2, as a global health emergency on 30 January 2020 and as a pandemic on 11 March 2020 [1]. This disease is known for its rapid progression to severe clinical manifestations, frequently causing complications such as sepsis, respiratory failure, acute respiratory distress syndrome and death [2]. Severe COVID-19 disease is postulated to happen due to the overproduction of proinflammatory cytokines (interleukin (IL)-1, IL-6 and tumour necrosis factor) causing a cytokine storm syndrome [3, 4]. However, the lack of knowledge and absence of definitive treatment for COVID-19 has brought a considerable toll on healthcare systems globally [5, 6].

Currently, COVID-19 has caused health equipment shortages worldwide, and the allocation of scarce resources is problematic and presents with many ethical problems [7, 8]. In order to provide the most benefit to COVID-19 patients, there is an increasing need to better allocate these scarce resources. Raised D-dimer level is considered a poor prognostic feature for COVID-19 patients [9]. More recently, according to a pooled analysis study, increased D-dimer values were frequently found in patients with a severe COVID-19, suggesting that this biomarker has a promising potential for determining mortality [10]. Few studies have also reported an increased risk of mortality in COVID-19 patients with elevated on-admission D-dimer levels [11, 12].

To date, no systematic review and meta-analysis has been carried out to gather the existing evidence of using on-admission D-dimer levels to evaluate the all-cause mortality risk in COVID-19 patients. Therefore, we aim to review and evaluate currently available evidence to establish the association between D-dimer levels on admission and all-cause mortality risk in patients with COVID-19.

## Methods

### Protocol and registration

This systematic review and meta-analysis was written according to the Preferred Reporting Items for Systematic Reviews and Meta-Analyses (PRISMA) Checklist (Table S1). Previously, the protocol for this systematic review was registered in the International Prospective Register of Systematic Reviews (PROSPERO) database on 20 May 2020 (CRD42020186616).

### Search strategy

We performed a systematic literature search for peer-reviewed papers published from database conception to 24 May 2020 in four databases (Ovid MEDLINE, EMBASE, SCOPUS and Web of Science databases). To gather the most recent and accurate data, we also searched for preprints from two databases (MedRxiv and SSRN), and for grey literature from two databases (WHO COVID-19 Global Research Database and Center for Disease Control and Prevention COVID-19 Research Article). The combination of keywords (and its synonyms) included ‘COVID-19’, ‘D-dimer’ and ‘Mortality’, and were adapted to each respective database (Table S2). Forward and backward tracing of references from relevant articles and manual handsearching were performed to identify additional papers potentially missed in the databases.

### Eligibility criteria

Due to time restrictions and lack of resources, we only included papers published in English. We included studies on adult (≥18 years old) COVID-19 patients that reported D-dimer levels on admission as a categorical variable (with any cut-off values), and all-cause mortality risk expressed in hazard ratio (HR), risk ratio (RR) or odds ratio (OR). Studies must be able to provide the HR, RR or OR, or, at the very least, sufficient raw data allowing reanalysis with the two-by-two contingency table. Based on the hierarchy of evidence, cohort studies were included. However, to include the most comprehensive evidence, we also included case series with sufficient data to conduct a reanalysis as cohort studies [13].

### Study selection, data extraction and risk of bias assessment

All articles retrieved were exported and stored in Endnote X9. After removing duplicates, the titles and abstracts were screened by two independent reviewers (D.M.S. and A.M.L.), and full-text screening of articles was done using the eligibility criteria. Any uncertainties regarding the study selection were discussed until a common agreement was reached.

A standardised extraction form was used to extract the data from all included studies. The extracted data included information on: first author, publication date, study location, study period, study design, baseline population characteristics (number of patients, age, gender and presence of co-morbidities such as hypertension, diabetes and cardiovascular diseases), exposure (D-dimer levels on admission presented as categorical variable with any cut-off values) and outcome (all-cause mortality).

All included studies were then assessed for its quality and risk of bias by two independent reviewers (D.M.S. and A.M.L.). Any discrepancies regarding the assessment were discussed until an agreement was reached. Cohort studies and case series were assessed using the Newcastle-Ottawa Scale (NOS) and the Joanna Briggs Institute (JBI) Critical Appraisal Tool for Case Series, respectively.

### Data synthesis and statistical analysis

We then exported the data from all studies to Review Manager software (RevMan 5.3) and performed quantitative synthesis in a meta-analysis. The heterogeneity of the selected studies was determined using Cochrane chi-square and *I*^2^. The Mantel–Haenszel method was used to summarise the data between the studies and calculate the pooled RR with the 95% confidence interval (CIs). The random-effect model (DerSimonian and Laird method) was used if there were significant heterogeneity (*I*^2^ > 50%) and the fixed-effect model was used if *I*^2^ ≤ 50%. The potential publication bias of the included studies was assessed visually using a funnel plot comparing the RR with the standard error of the natural log of RR. Sensitivity analysis was performed by omitting one study at a time, by restricting the analysis to studies with 0.5 μg/ml as the D-dimer cut-off value, to only cohort studies, and to only peer-reviewed studies. Subgroup analysis was performed to compare the results based on the study location (Chinese and non-Chinese). A statistically significant finding was considered when a two-tailed *P* < 0.05.

## Results

### Search selection and quality assessment

**Fig. 1.**
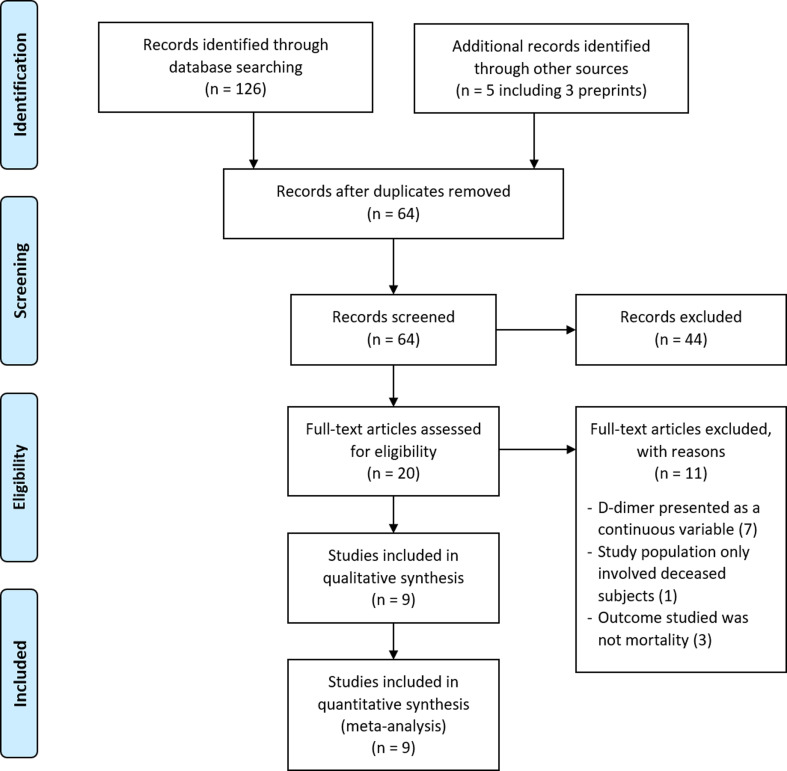
PRISMA diagram showing the study selection for inclusion in the meta-analysis. Literature search identifying peer-reviewed papers, preprints and grey literature was done from database conception to 24 May 2020.

A comprehensive search was performed according to the prespecified search strategy. Peer-reviewed papers from database searching resulted in 126 papers, and additional searching from other sources, including preprints, grey literatures and manual handsearching, resulted in five additional papers. After removing duplicate records, 64 unique articles were screened for its title and abstract, and the remaining 20 full-text articles were assessed for its eligibility. A total of nine papers, three of which were preprints, met the inclusion criteria and were subsequently reviewed (Fig. 1). Overall, there were six cohort studies [11, 14–18] and two case series [19, 20] comparing on-admission D-dimer levels between non-survivors and survivors, and one cohort study [12] comparing patients with elevated D-dimer levels and normal D-dimer levels on admission. Only two studies were prospective studies [14, 18], while the others were retrospective in nature. All studies were conducted in China [11, 12, 14–17, 19], except for two studies carried out in the USA [20] and Italy [18], respectively. The quality and risk of bias assessment of the included studies are shown in Tables S3 and S4. In brief, all cohort studies were of acceptable quality; with a score of 7 and 8. Additionally, all included case series had high scores (scores of 9 and 10) according to the JBI Critical Appraisal Tool.

### Baseline characteristics and study findings

The summary of baseline characteristics and study findings are presented in Table 1. This systematic review and meta-analysis included a total of 2911 patients (2223 survivors and 688 non-survivors), the majority of which were male (53.8%). The non-survivor group was generally older than the survivor group among all the included studies and was more likely to have underlying diseases (such as hypertension, diabetes and cardiovascular diseases) upon admission compared to the survivor groups. Different cut-off values for D-dimer were used; four studies used 0.5 μg/ml [11, 14, 15, 18], two studies used 2.0 μg/ml [12, 20], and the remaining three studies used 21 μg/ml [19], 1 μg/ml [16] and 0.55 μg/ml [17] respectively. None of the studies provided their reasons for choosing such cut-off values except for Zhang *et al*., which determined the optimal D-dimer cut-off of 2.0 μg/ml curve (with a sensitivity of 92.3% and specificity of 83.3%) by using a receiver operator characteristic (ROC) [12].

**Table 1.** Summary of baseline characteristics from studies included in the meta-analysis

Author	Publication date	Study location	Study period	Study design	Cut-off values (μg/ml)	Groups	Number of participants		Age (years)	*P*	HT, *n* (%)	*P*	Diabetes, *n* (%)	*P*	CVD, *n* (%)	*P*
							*n*	M/F								
Peer-reviewed																
Zhou F	09/03/20	Jinyintan Hospital and Wuhan Pulmonary Hospital, Wuhan, China	Dec 29, ’19 to Jan 31, ’20	Retrospective cohort study	0.5	Survivor	137	81/56	52	<0.0001	32 (23)	0.0008	19 (14)	0.0051	2 (1)	<0.0001
						Non-survivor	54	38/16	69		26 (48)		17 (31)		13 (24)	
Chen T	17/03/20	Tongji Hospital, Wuhan, China	Jan 13, ’20 to Feb 28, ’20	Retrospective case series	21.0	Survivor	161	73/88	51	NR	39 (24)	NR	23 (14)	NR	7 (4)	NR
						Non-survivor	113	30/83	68		54 (48)		24 (21)		16 (14)	
Du R	30/03/20	Wuhan Pulmonary Hospital, China	Dec 25, ’19 to Feb 7, ’20	Prospective cohort study	0.5	Survivor	158	87/71	56	<0.001	45 (29)	0.005	27 (17)	0.231	17 (11)^a^	<0.001
						Non-survivor	21	10/11	70		13 (62)		6 (29)		12 (57)^a^	
Cao J	02/04/20	Wuhan University Zhongnan Hospital, Wuhan, China	Jan 3, ’20 to Feb 15, ’20	Retrospective cohort study	0.5	Survivor	85	40/45	53	<0.001	17 (20)	<0.001	5 (6)	<0.001	2 (2)	0.040
						Non-survivor	17	13/4	72		11 (65)		6 (25)		3 (18)	
Zhang L	19/04/20	Wuhan Asia General Hospital, Wuhan, China	Jan 12, ’20 to Mar 15, ’20	Retrospective cohort study	2.0	Overall	343	170/174	62	N/A	76 (22)	N/A	47 (14)	N/A	19 (6)	N/A
Yao Q	24/04/20	Dabieshan Medical Center, Hubei, China	Jan 30, ’20 to Mar 3, ’20	Retrospective cohort study	1.0	Survivor	96	NS: 30/53 S: 6/7	NS: 50 S: 56	<0.001	NS: 7 (8) S: 2 (15)	0.001	NS: 2 (2) S: 2 (15)	0.166	NS: 2 (2) S: 0 (0)	NR
						Non-survivor	12	7/5	65		7 (58)		1 (8)		2 (17)	
Preprints																
Luo X	19/03/20	Eastern Campus of Renmin Hospital, Wuhan, China	Jan 30, ’20 to Feb 25, ’20	Retrospective cohort study	0.55	Survivor	303	136/167	49	<0.001	53 (18)	<0.001	32 (11)	<0.001	20 (7)	0.004
						Non-survivor	100	57/43	71		60 (60)		25 (25)		16 (16)	
Paranjpe I	19/04/20	5 hospitals, New York, USA	Feb 27, ’20 to Apr 2, ’20	Case series	2.0	Survivor	768	436/332	59	NR	233 (30)	NR	151 (20)	NR	137 (18)	NR
						Non-survivor	310	191/119	75		140 (45)		105 (34)		147 (47)	
Giacomelli A	02/05/20	Luigi Sacco Hospital, Milan, Italy	Feb 21, ’20 to Apr 20, ’20	Prospective cohort study	0.5	Survivor	185	122/63	NR	NR	NR	NR	NR	NR	NR	NR
						Non-survivor	48	39/9	NR		NR		NR		NR	

CVD, cardiovascular disease; HT, hypertension; M/F, male/female; *n*, number; N/A, not applicable; NR, not reported; NS, non-severe; *P*, *P*-value; s, severe.

*Statistically significant result, P* < 0.05.

a Includes cerebrovascular diseases.

### Association between elevated D-dimer levels on admission and all-cause mortality risk

**Fig. 2.**
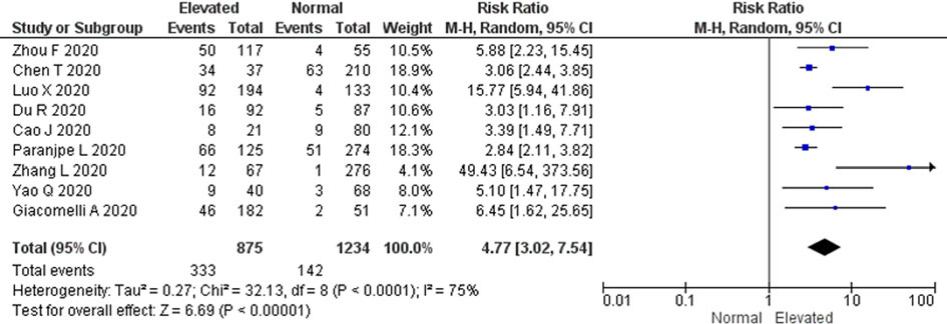
All-cause mortality risk. Forest plot using the Mantel–Haenszel random-effect model demonstrating the association between D-dimer levels on admission and all-cause mortality risk for all included studies.

There were a total of 475 non-survivors and 1634 survivors with available D-dimer levels on admission from all included studies in this meta-analysis. The pooled RR for all-cause mortality in elevated D-dimer levels on admission, regardless of the cut-off used, was 4.77 (95% CI 3.02–7.54). There was a significant heterogeneity among the studies (*P* < 0.0001; *I*^2^ = 75%) (Fig. 2). The funnel plot revealed no publication bias (Fig. S1). Sensitivity analysis by sequentially removing one study at a time showed no significant changes to the pooled RR and the conclusion of the results. Further restriction of the analysis to only cohort studies and only peer-reviewed studies did not significantly change the conclusion of the primary analysis and resulted in pooled RR values for all-cause mortality of 6.45 (95% CI 3.45–12.05) and 4.43 (95% CI 4.43–7.92), respectively (Figs S2 & S3).

### All-cause mortality risk among studies with D-dimer cut-off value of 0.5 μg/ml

**Fig. 3.**
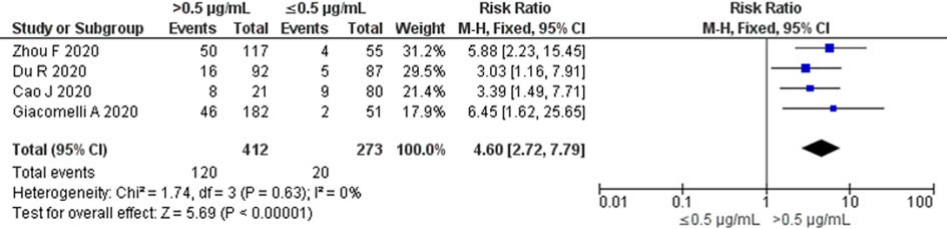
All-cause mortality risk for studies with D-dimer cut-off value of 0.5 μg/ml. Forest plot using the Mantel–Haenszel fixed-effect model showing the association between D-dimer levels on admission and all-cause mortality risk for studies with D-dimer cut-off value of 0.5 μg/ml.

No significant heterogeneity among the four available studies with 0.5 μg/ml as the cut-off value was found when we restricted our analysis based on a single cut-off value (*P* = 0.63; *I*^2^ = 0%) (Fig. 3). The pooled RR for all-cause mortality using the Mantel–Haenszel fixed-effect model was 4.60 (95% CI 2.72–7.79) in elevated D-dimer levels *vs.* normal D-dimer levels on admission.

### All-cause mortality risk based on study location

**Fig. 4.**
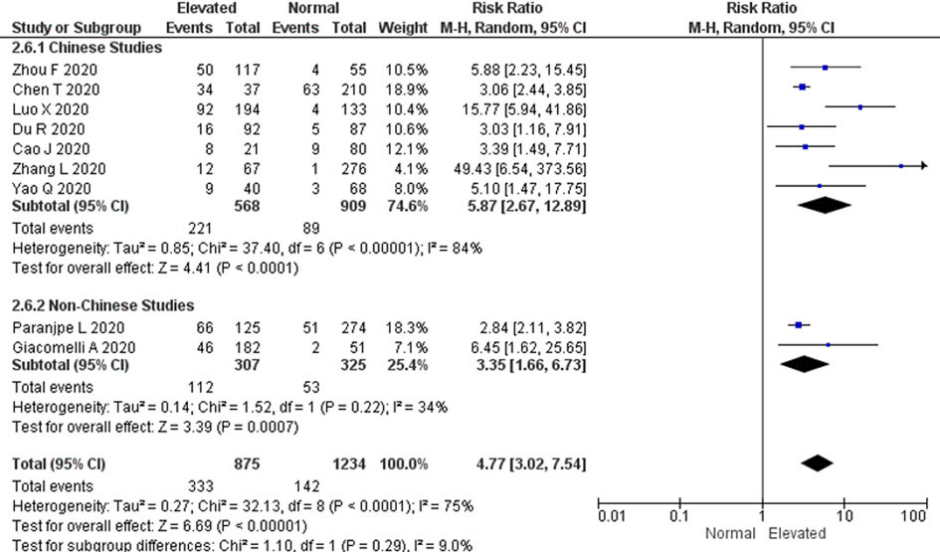
Subgroup analysis by study location. Forest plot using the Mantel–Haenszel random-effect model comparing the association between D-dimer levels on admission and all-cause mortality risk in Chinese and non-Chinese studies. Non-Chinese studies included studies done in the USA and Italy.

To compensate for the differences in D-dimer levels influenced by ethnicity, we attempted to perform subgroup analysis by study location (Chinese and non-Chinese studies) [21, 22]. The subgroup analysis showed that the pooled RR for all-cause mortality was higher in Chinese studies *vs.* non-Chinese studies (RR 5.87; 95% CI 2.67–12.89 and RR 3.35; 95% CI 1.66–6.73, respectively), however this was not statistically significant (*P* = 0.29). A significant heterogeneity among the Chinese studies (*P* < 0.00001; *I*^2^ = 84%) was also observed but was not found among non-Chinese studies (*P* = 0.22; *I*^2^ = 34%) (Fig. 4).

## Discussion

The result of this comprehensive meta-analysis, which included a total of 2911 COVID-19 patients with an established outcome of survivor and non-survivor, showed that, in all included studies, elevated D-dimer levels on admission were significantly associated with a greater risk of in-hospital all-cause mortality. This was observed regardless of the different cut-off values used to determine an elevated D-dimer level among studies. Overall, the pooled mortality RR was roughly five times in elevated D-dimer level *vs.* normal D-dimer level on admission. Sensitivity analysis did not significantly change the conclusion of the result. Additionally, restricting the analysis to only studies with 0.5 μg/ml as the cut-off value showed a similar pooled mortality risk to the primary analysis. Furthermore, subgroup analysis showed that all-cause mortality risk was higher in Chinese studies compared to non-Chinese studies, despite no statistical significance.

Previously, elevated D-dimer levels have been shown to predict poor prognosis in patients with CAP [23]. Studies have also reported that D-dimer levels on admission were more frequently found in patients with severe COVID-19 cases [9, 10, 24]. The result of our meta-analysis showed that COVID-19 patients with elevated on-admission D-dimer levels have a significantly higher risk of all-cause mortality. D-dimer is a fragment produced by the cleavage of fibrin by plasmin during clot breakdown [25]. Therefore, a high on-admission D-dimer could suggest increased fibrinolysis, some evidence of intravascular coagulation and thrombotic disease, and indicate cytokine storm, tissue damage or potentially the occurrence of sepsis as seen in the severe clinical manifestation of COVID-19 [26, 27]. Recent evidences have also shown that patients with severe COVID-19 have an increased incidence of pulmonary embolism and deep vein thrombosis during the clinical course of the disease [28–30].

Currently, there has yet to be a common consensus on the D-dimer threshold level for predicting the prognosis of COVID-19 patients, and thus, different studies have utilised different D-dimer cut-off levels [31, 32]. Unfortunately, none of the studies, except for Zhang *et al*. reported the reasoning for selecting such cut-off levels. Zhang *et al*. used the ROC curve to establish its cut-off value of 2.0 μg/ml, which supported the International Society of Thrombosis and Haemostasis (ISTH) arbitrary definition of raised D-dimers on admission in COVID-19 as three–four-fold increase [12, 31]. We noted that significant heterogeneity between studies was observed when including all available studies and disregarding the use of a specific cut-off value. However, restricting analysis to studies with cut-off value of 0.5 μg/ml, despite showing no significant heterogeneity between studies, showed a similar pooled RR for all-cause mortality. Based on our findings, we suggest that a cut-off value of 0.5 μg/ml can be used to identify the mortality risk of COVID-19 patients upon admission. However, determining the optimal threshold value without statistical evaluation is problematic as altering threshold values would subsequently result in changes of sensitivity and specificity of the biomarker [33]. Therefore, further studies should consider using a specific and common cut-off value for D-dimer, especially for COVID-19 patients.

The prediction of all-cause mortality using on-admission D-dimer value requires careful interpretation. Baseline D-dimer levels are influenced by patient characteristics, such as age, ethnicity, active malignancy, immobility and prior thromboembolic diseases [21, 22, 34]. We noted that in comparison with the survivors, non-survivors of COVID-19 patients were generally much older and presented more frequently with underlying diseases of hypertension, diabetes and cardiovascular diseases. Unfortunately, adjustments for these variables in our analysis, which are potential confounders to the study, were not possible. Thus, we could not exclude the possibility that older patients and the presence of underlying diseases such as hypertension, cardiovascular diseases and diabetes, could influence the association between D-dimer levels and all-cause mortality risk. Analysis by study location also showed variations in all-cause mortality risk between Chinese studies and non-Chinese studies, suggesting the need for different cut-offs between countries.

As of the writing of this meta-analysis, there has yet to be a meta-analysis of studies investigating the all-cause mortality risk in COVID-19 patients with elevated and normal D-dimer levels. Our search strategy attempted to retrieve all available evidence from peer-reviewed papers, preprints and grey literature, and in doing so, have identified studies that were conducted outside of China. We also performed a robust statistical analysis, with sensitivity and subgroup analysis, and showed no significant changes to the overall conclusion of the result.

However, we acknowledge several limitations to this meta-analysis. First, most of the studies were from China, whereas currently the most confirmed cases and deaths are found in the USA and Europe. Second, we are aware that restriction of included studies to only English would result in potential bias. Third, most studies were retrospective in nature and were more prone to bias and frequent loss of data compared to prospective cohort studies. Finally, the threshold D-dimer values used vary between the included studies and the selected D-dimer reference value was only evaluated and statistically determined in one study [12].

## Conclusion

D-dimer level on admission has a promising prognostic value for predicting all-cause mortality of COVID-19 patients. Despite the differences in threshold values across the studies, there was a roughly fivefold increase in all-cause mortality for patients with elevated D-dimer levels on admission compared to normal level. Although our meta-analysis has shown that determination of mortality risk with a D-dimer cut-off value of 0.5 μg/ml is possible, a consensus cut-off for D-dimer concentration must be established before clinical implementation. We recommend more studies, from different geographic locations, are to be conducted to assess the prognostic value of D-Dimer level on admission among different ethnicities. Furthermore, future meta-analysis of individual participant data, and with statistical adjustments for potential confounders, are needed to confirm our findings.

## Data Availability

The datasets used for this meta-analysis are available on request to the corresponding author.

## Supplementary material

For supplementary material accompanying this paper visit https://doi.org/10.1017/S0950268820002022. click here to view supplementary material
