# Supplementary material for: D-dimer levels on admission and all-cause mortality risk in COVID-19 patients: a meta-analysis
Source: Epidemiol Infect. 2020 Sep 7;148:e202. doi: 10.1017/S0950268820002022 (PMC7487805; doi:10.1017/S0950268820002022)

**List of Supplementary Materials**

**Table S1. PRISMA checklist**

**Table S2. Search strategy used in peer-reviewed databases**

**Table S3. Risk of bias assessment of cohort studies included in the meta-analysis using the Newcastle-Ottawa Scale (NOS)**

**Table S4. Risk of bias assessment of case series included in the meta-analysis using the Joanna Briggs Institute Critical Appraisal Tool**

**Figure S1. Publication bias assessment of all studies included in the meta-analysis**

**Figure S2. Sensitivity analysis restricted to only cohort studies**

**Figure S3. Sensitivity analysis restricted to only peer-reviewed studies**

**Table S1. PRISMA checklist**

| **Section/topic** | **#** | **Checklist item** | **Reported on page #** |
| --- | --- | --- | --- |
| **TITLE** | | |  |
| Title | 1 | Identify the report as a systematic review, meta-analysis, or both. | 1 |
| **ABSTRACT** | | |  |
| Structured summary | 2 | Provide a structured summary including, as applicable: background; objectives; data sources; study eligibility criteria, participants, and interventions; study appraisal and synthesis methods; results; limitations; conclusions and implications of key findings; systematic review registration number. | 2, 3 |
| **INTRODUCTION** | | |  |
| Rationale | 3 | Describe the rationale for the review in the context of what is already known. | 4 |
| Objectives | 4 | Provide an explicit statement of questions being addressed with reference to participants, interventions, comparisons, outcomes, and study design (PICOS). | 4 |
| **METHODS** | | |  |
| Protocol and registration | 5 | Indicate if a review protocol exists, if and where it can be accessed (e.g., Web address), and, if available, provide registration information including registration number. | 5 |
| Eligibility criteria | 6 | Specify study characteristics (e.g., PICOS, length of follow-up) and report characteristics (e.g., years considered, language, publication status) used as criteria for eligibility, giving rationale. | 5, 6 |
| Information sources | 7 | Describe all information sources (e.g., databases with dates of coverage, contact with study authors to identify additional studies) in the search and date last searched. | 5 |
| Search | 8 | Present full electronic search strategy for at least one database, including any limits used, such that it could be repeated. | 5, Supporting Information |
| Study selection | 9 | State the process for selecting studies (i.e., screening, eligibility, included in systematic review, and, if applicable, included in the meta-analysis). | 6 |
| Data collection process | 10 | Describe method of data extraction from reports (e.g., piloted forms, independently, in duplicate) and any processes for obtaining and confirming data from investigators. | 6 |
| Data items | 11 | List and define all variables for which data were sought (e.g., PICOS, funding sources) and any assumptions and simplifications made. | 6 |
| Risk of bias in individual studies | 12 | Describe methods used for assessing risk of bias of individual studies (including specification of whether this was done at the study or outcome level), and how this information is to be used in any data synthesis. | 6 |
| Summary measures | 13 | State the principal summary measures (e.g., risk ratio, difference in means). | 5, 6, 7 |
| Synthesis of results | 14 | Describe the methods of handling data and combining results of studies, if done, including measures of consistency (e.g., I^2^) for each meta-analysis. | 6, 7 |

| **Section/topic** | **#** | **Checklist item** | **Reported on page #** |
| --- | --- | --- | --- |
| Risk of bias across studies | 15 | Specify any assessment of risk of bias that may affect the cumulative evidence (e.g., publication bias, selective reporting within studies). | 6,7 |
| Additional analyses | 16 | Describe methods of additional analyses (e.g., sensitivity or subgroup analyses, meta-regression), if done, indicating which were pre-specified. | 7 |
| **RESULTS** | | |  |
| Study selection | 17 | Give numbers of studies screened, assessed for eligibility, and included in the review, with reasons for exclusions at each stage, ideally with a flow diagram. | 8, 18 |
| Study characteristics | 18 | For each study, present characteristics for which data were extracted (e.g., study size, PICOS, follow-up period) and provide the citations. | 8, 9, 17 |
| Risk of bias within studies | 19 | Present data on risk of bias of each study and, if available, any outcome level assessment (see item 12). | 8, Supporting Information |
| Results of individual studies | 20 | For all outcomes considered (benefits or harms), present, for each study: (a) simple summary data for each intervention group (b) effect estimates and confidence intervals, ideally with a forest plot. | 8-10, Supporting Information |
| Synthesis of results | 21 | Present results of each meta-analysis done, including confidence intervals and measures of consistency. | 8-10, 19 Supporting Information |
| Risk of bias across studies | 22 | Present results of any assessment of risk of bias across studies (see Item 15). | 9, Supporting Information |
| Additional analysis | 23 | Give results of additional analyses, if done (e.g., sensitivity or subgroup analyses, meta-regression [see Item 16]). | 9, 10, 20, 21, Supporting Information |
| **DISCUSSION** | | |  |
| Summary of evidence | 24 | Summarize the main findings including the strength of evidence for each main outcome; consider their relevance to key groups (e.g., healthcare providers, users, and policy makers). | 11, 12 |
| Limitations | 25 | Discuss limitations at study and outcome level (e.g., risk of bias), and at review-level (e.g., incomplete retrieval of identified research, reporting bias). | 13 |
| Conclusions | 26 | Provide a general interpretation of the results in the context of other evidence, and implications for future research. | 14 |
| **FUNDING** | | |  |
| Funding | 27 | Describe sources of funding for the systematic review and other support (e.g., supply of data); role of funders for the systematic review. | 15 |

*From:*  Moher D, Liberati A, Tetzlaff J, Altman DG, The PRISMA Group (2009). Preferred Reporting Items for Systematic Reviews and Meta-Analyses: The PRISMA Statement. PLoS Med 6(7): e1000097. doi:10.1371/journal.pmed1000097

**Table S2. Search strategy used in peer-reviewed databases.** Results showed database search done from database conception to 24 May 2020.

| **Database** | **Search Terms** | **Hits** |
| --- | --- | --- |
| Ovid MEDLINE | 1. Covid-19 2. “Coronavirus 2019” 3. 2019-nCoV 4. SARS-CoV-2 5. 1 OR 2 OR 3 OR 4 6. D-dimer 7. Mortality 8. Death 9. Non-survivor 10. 7 OR 8 OR 9 11. 5 AND 6 AND 10 | 40 |
| EMBASE | ('covid-19' OR 'coronavirus 2019' OR '2019-ncov' OR 'sars-cov-2') AND 'd-dimer' AND (mortality OR death OR ‘non-survivor’) | 39 |
| SCOPUS | (TITLE-ABS-KEY(covid-19) OR TITLE-ABS-KEY("coronavirus 2019") OR TITLE-ABS-KEY(2019-ncov) OR TITLE-ABS-KEY(sars-cov-2)) AND TITLE-ABS-KEY(d-dimer) AND (TITLE-ABS-KEY(mortality) OR TITLE-ABS-KEY(death) OR TITLE-ABS-KEY(non-survivor)) | 39 |
| Web of Science databases | ALL=((Covid-19 OR "coronavirus 2019" OR 2019-nCoV OR SARS-CoV-2) AND D-Dimer AND (death OR mortality OR non-survivor)) | 8 |
| **Total** |  | **126** |

**Table S3.** **Risk of bias assessment of cohort studies included in the meta-analysis using the Newcastle-Ottawa Scale (NOS)**

| **Author** | **Publication Date** | **Publication Type** | **Selection** | **Comparability** | **Outcome** | **Total** |
| --- | --- | --- | --- | --- | --- | --- |
| Zhou F | 09/03/2020 | Peer-reviewed | ****** | ****** | ******* | **7/9** |
| Yao Q | 24/04/2020 | Peer-reviewed | ****** | ****** | ******* | **7/9** |
| Du R | 30/03/2020 | Peer-reviewed | ****** | ****** | ******* | **7/9** |
| Cao J | 02/04/2020 | Peer-reviewed | ****** | ****** | ******* | **7/9** |
| Zhang L | 19/04/2020 | Peer-reviewed | ****** | ****** | ******* | **7/9** |
| Luo X | 19/03/2020 | Preprints | ****** | ****** | ******* | **7/9** |
| Giacomelli A | 02/05/2020 | Preprints | ******* | ****** | ******* | **8/9** |

**Table S4. Risk of bias assessment of case series included in the meta-analysis using the Joanna Briggs Institute Critical Appraisal Tool**

| **Author** | **Publication Date** | **Publication Type** | **Q1** | **Q2** | **Q3** | **Q4** | **Q5** | **Q6** | **Q7** | **Q8** | **Q9** | **Q10** | **Total** |
| --- | --- | --- | --- | --- | --- | --- | --- | --- | --- | --- | --- | --- | --- |
| Chen T | 17/03/2020 | Peer-reviewed | **Y** | **Y** | **Y** | **Y** | **Y** | **Y** | **Y** | **Y** | **Y** | **Y** | **10/10** |
| Paranjpe L | 19/04/2020 | Preprints | **Y** | **Y** | **Y** | **Y** | **Y** | **Y** | **Y** | **Y** | **Y** | **N** | **9/10** |

**Figure S1. Publication bias assessment of all studies included in the meta-analysis.** Funnel Plot showing all included studies. RR = Risk Ratio, SE(log[RR]) = Standard Error of the natural log of RR. A = Zhou F et al., B = Chen T et al., C = Guo X et al., D = Du R et al., E = Cao J et al., F = Paranjpe L et al., G = Zhang L et al., H = Yao Q et al., I = Giacomelli A et al.

**
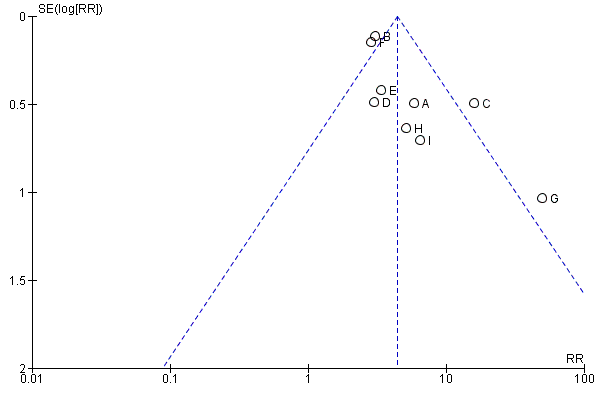
**

**Figure S2. Sensitivity analysis restricted to only cohort studies.** Forest Plot using the Mantel-Haenszel random-effect model showing the association between D-dimer levels on admission and all-cause mortality in included cohort studies.


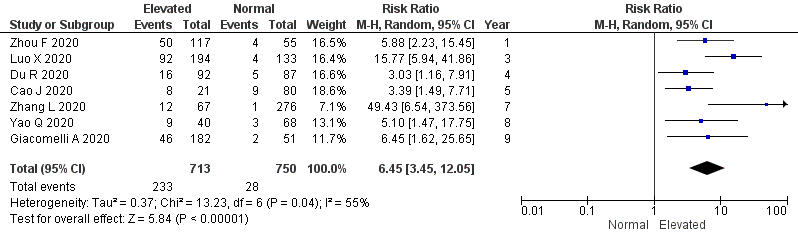

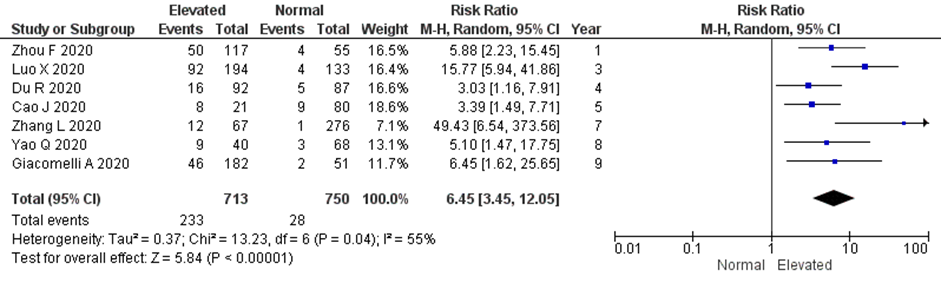


**Figure S3. Sensitivity analysis restricted to only peer-reviewed studies.** Forest Plot using the Mantel-Haenszel random-effect model showing the association between D-dimer levels on admission and all-cause mortality in included peer-reviewed studies.


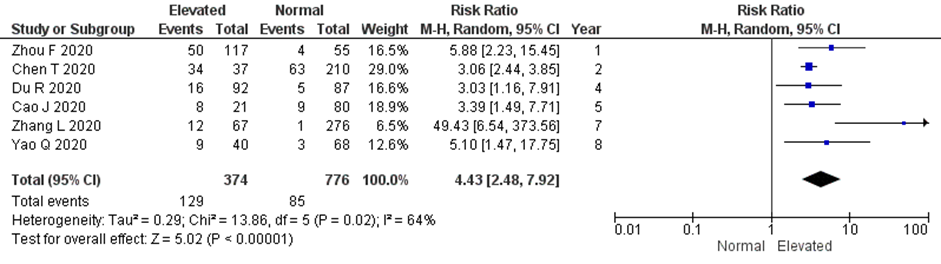

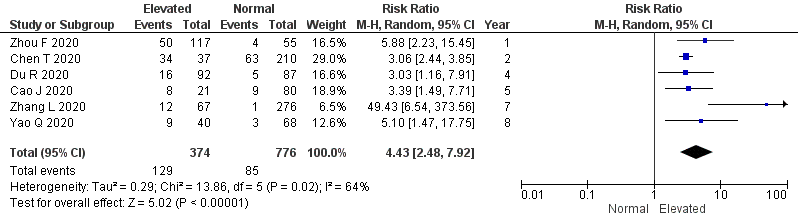

Supplement: Supplementary file 1 [file S0950268820002022sup.zip › S0950268820002022sup004.docx]
