# Supplementary figures and images for: D-dimer levels on admission and all-cause mortality risk in COVID-19 patients: a meta-analysis
Source: Epidemiol Infect. 2020 Sep 7;148:e202. doi: 10.1017/S0950268820002022 (PMC7487805; doi:10.1017/S0950268820002022)

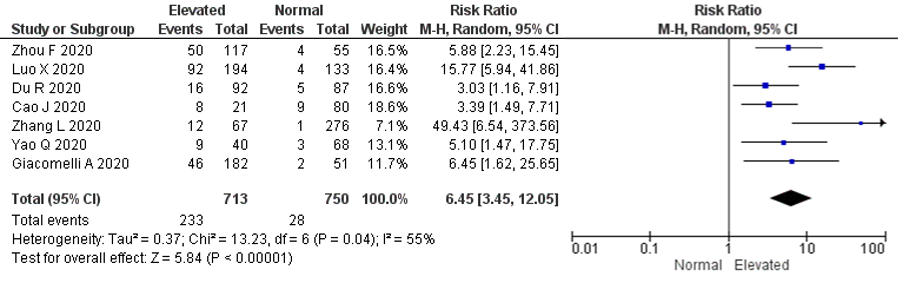

Supplement: Supplementary file 1 [file S0950268820002022sup.zip › S0950268820002022sup001.tif]

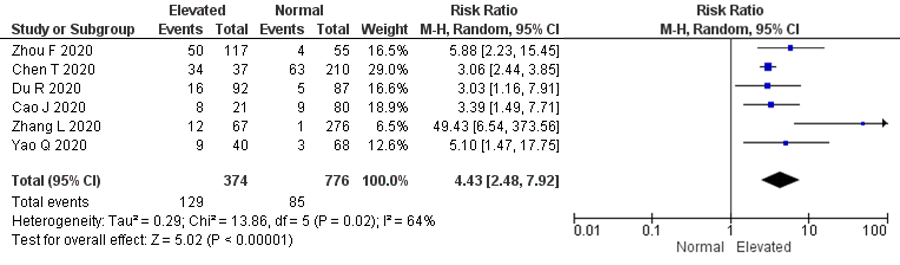

Supplement: Supplementary file 1 [file S0950268820002022sup.zip › S0950268820002022sup002.tif]

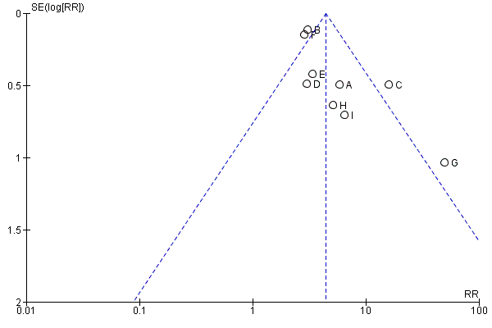

Supplement: Supplementary file 1 [file S0950268820002022sup.zip › S0950268820002022sup003.tif]
